# A Novel Line Space Voting Method for Vanishing-Point Detection of General Road Images

**DOI:** 10.3390/s16070948

**Published:** 2016-06-23

**Authors:** Zongsheng Wu, Weiping Fu, Ru Xue, Wen Wang

**Affiliations:** 1 School of Mechanical and Precision Instrumental Engineering, Xi’an University of Technology, Xi’an 710048, China; wuzs2005@163.com (Z.W.); wangwen@xaut.edu.cn (W.W.); 2 School of Information Engineering, Xizang Minzu University, XianYang 712082, China; joywinjam@163.com

**Keywords:** vanishing point detection, texture orientation, LSD, line space voting

## Abstract

Vanishing-point detection is an important component for the visual navigation system of an autonomous mobile robot. In this paper, we present a novel line space voting method for fast vanishing-point detection. First, the line segments are detected from the road image by the line segment detector (LSD) method according to the pixel’s gradient and texture orientation computed by the Sobel operator. Then, the vanishing-point of the road is voted on by considering the points of the lines and their neighborhood spaces with weighting methods. Our algorithm is simple, fast, and easy to implement with high accuracy. It has been experimentally tested with over hundreds of structured and unstructured road images. The experimental results indicate that the proposed method is effective and can meet the real-time requirements of navigation for autonomous mobile robots and unmanned ground vehicles.

## 1. Introduction

**Figure 1:**
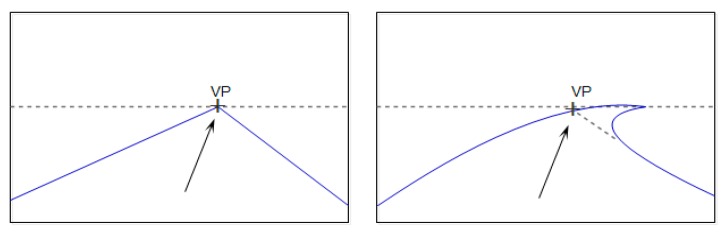
Vanishing point (VP) in the straight road (**left**) and curved road (**right**).

As computer vision techniques are further developing, visual perception is becoming widely applied to the autonomous navigation systems (ANSs) for mobile robots and unmanned ground vehicles. In these ANSs, the location of an estimated vanishing point can guide the driving direction and help it to detect the road area. A set of straight parallel lines in the world space when viewed by perspective projection converge to a common point in the image space known as the vanishing point (VP), which can usually be used to guide the mobile robot or automated vehicle forward [1]. Figure 1 shows the role of the VP in an intelligent navigation system. The cross point denotes the VP, and the arrow represents the driving direction. Whether in considering a straight or curved road, the vanishing point may be the end of the current road. It can be utilized to steer the vehicle or used as a directional constraint for drivable region segmentation.

In recent years, many texture-based vanishing-point methods [2,3,4,5,6,7,8] have been presented. They look for a global road constraint to distinguish road direction instead of searching for locally distinctive road cues. Texture-based methods look for local oriented textures of the pixels and then consider them for the basis of voting for the locations of the road’s vanishing points. The location with the maximum number of votes is regarded as the vanishing point of the main road region. Moreover, the direction of the road and the road boundaries can be extracted by the vanishing-point location and texture orientation. These methods can obtain the correct position of the VP, but they require a lot of time to be spent on voting for the VP. 

In order to reduce the computational time, we propose a novel fast vanishing-point detection method for autonomous navigation systems in structured and unstructured road terrains in this paper. We use the line segment detector (LSD) method to extract the line segments from the road image and extend the valid line segments to the boundary of the image. Then, the points on the lines and their neighborhood points vote for the vanishing point in the corresponding accumulator space. In the voting process, a length weight, orientation weight, and Gaussian space weight of the voting points are introduced to control the voting scores. The point having the highest score in the accumulator space is considered to be the estimated vanishing point of the road. This method is called a line space voting method. Due to the low computational complexity, our proposed method is very efficient. 

The remainder of this paper is organized as follows: Section 2 describes related algorithms of vanishing-point detection. A novel line space voting method with weighting methods for robust vanishing-point estimation is presented in Section 3. Section 4 provides the experimental results. Finally, our conclusions are mentioned in Section 5.

## 2. Related Work

Generally, edge-based vanishing-point detection methods [9,10] are unsuitable for the most general sort of roads, especially when the road has a little color difference between its surface and the surrounding environment, or when the road has no remarkable boundaries or markings. Instead, the texture-based methods [2,8] are more effective at detecting the road region for general road images. Rasmussen [3] proposed a texture-based method to replace the noise-sensitive edge detection step of vanishing-point detection algorithms. Kong et al. [2,8] presented an effective vanishing-point detection method in which Gabor filter banks [11] are used to compute the dominant texture orientation at every pixel of the road image. In this method, a novel local adaptive soft voting (LASV) scheme based on variable-sized voting regions using confidence-weighted Gabor filters was proposed in order to determine the remaining voters by checking the reliability of the obtained texture orientations in the voting region. Moreover, a new vanishing point-constrained edge detection technique was proposed to search for road boundaries. However, the computational cost is high because of a large number of scanning pixels. In addition, an estimation error is prone to occur in some images, in which the radius of the proposed half-disk voting region is not large enough. Bui et al. [4,5,6] proposed a texture-based local soft voting method to overcome the limitations of the LASV method by reducing the number of remaining voters and the scanning pixels for VP candidates. After that, the authors combined the texture orientations and color information to generate a histogram for estimating the two most dominant road borders. This method performs well in general road images, but the computational cost is still high. Moghadam et al. [7] propose a method to directly estimate the local dominant orientation based on the joint activity of four Gabor filters with orientations [0°, 45°, 90°, 135°] followed by an efficient and robust voting scheme. They use each ray drawn along a dominant orientation with adaptive weight to vote for the VP in the accumulator space. This method decreases the computational time for the dominant texture orientation estimation. However, this method is easily disturbed by noise and potentially leads to a detection error, especially in the road images which feature more trees, grass, and other random textures. Kong et al. [12] extended their work to reduce the computational amount. They proposed an efficient gLoG-based road-vanishing-point detection method by only using the dominant texture orientations estimated at a sparse set of salient microblob road regions, where the gLoG filter is used to detect these salient microblob areas and simultaneously estimate their dominant texture orientations. Fan et al. [13] proposed a framework for vanishing point detection based on the Modified Adaptive Soft Voting (MASV) scheme. This method performs faster than the complete-map based gLoG.

Although the accuracy of the above methods for vanishing point detection is guaranteed based on pixel-wise voting, it is expensive in terms of computational costs during the voting stage because each pixel can be regarded as both a voter and a vanishing point candidate. Most of these methods improved the performance by reducing the number of voters or vanishing point candidates. However, the computational cost of these methods still is high. Therefore, these methods cannot meet the real-time requirements of the automated vehicle. Our approach is very efficient with low computational complexity and high accuracy for structured and unstructured road images.

## 3. Methods

Since all parallel road borders or edges, lane lines, and even ruts and tire tracks left by previous vehicles on the road appear to converge into a single vanishing point, and their texture orientations point to a common point location, as such, we can analyze the road image and extract the line segments according to the consistent texture orientation. Because the extensional directions of the most extracted line segments point to the vanishing point, we can find out the vanishing point of the road using these line segments. So, a line space voting method is proposed in this paper. We use the LSD (line segment detector) method to detect the line segments in road images. However, there are some interferential line segments in the set of the line segments extracted by the LSD method. Therefore, we first remove the invalid line segments, and then extend the remaining line segments to the boundary of the image. After that, the points on the lines and their 5 × 5 neighborhood points vote for the vanishing point in the corresponding accumulator space. In the voting process, we introduced the length, orientation, and Gaussian space weight of the points to control the voting scores. Finally, the point having the highest score in the accumulator space is considered to be the estimated vanishing point of the road.

### 3.1. Line Segment Detection

In the line segment detection algorithms, the Hough transform method is widely used as a classical algorithm. However, the Hough transform method detects the line segments more slowly and is easily interfered with by the noise in the image. The other serious drawback is that the Hough method will cause a lot of error detection in the texture areas with high edge density, such as in areas where there are a lot of grass and tree leaves. Due to a lack of consideration regarding the direction of edge points, the line segments extracted by the Hough method are messy. Moreover, the Hough method needs to set the proper threshold parameters, such as the number of edge points constituting the line segment and the spacing and length of line segments. A method such as this that uses a fixed threshold of parameters will lead to many obvious false-positive and false-negative errors.

A LSD method is proposed by Grompone [14] which is a fast linear-time Line Segment Detector with a false detection control. It can quickly and accurately detect the line segments in the digital image without parameter tuning for each image. First, LSD uses the gradient and level-line information to detect the locally straight contours on images, which are zones of the image where the gray level is changing fast enough from dark to bright or in reverse. The level-line angle (i.e., the angle between the level-line and the horizontal direction) is computed as

(1) $$\theta_{LevelLine}=\arctan\left(\frac{g_{x}(x,y)}{-g_{y}(x,y)}\right)$$

(2) $$g_{x}(x,y)=\left[\begin{array}{ccc} -1 & 0 & 1\\ -2 & 0 & 2\\ -1 & 0 & 1 \end{array}\right]*I(x,y),\;g_{y}(x,y)=\left[\begin{array}{ccc} -1 & -2 & -1\\ 0 & 0 & 0\\ 1 & 2 & 1 \end{array}\right]*I(x,y)$$

where I(x,y) is the image gray level value at pixel (*x*, *y*) in the input image. $g_{x}(x,y)$ and $g_{y}(x,y)$ are the gradients of the pixel (*x*, *y*) in horizontal direction and vertical direction, respectively. The image gradient is computed with Equation (2) using the Sobel operator with 3 × 3 horizontal and vertical direction masks which replace the original 2 × 2 masks. The details of the LSD algorithm can be referred to in the literature [14].

**Figure 2:**
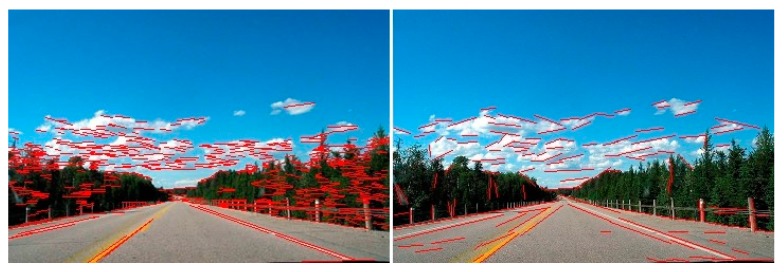
Comparison of line segment detection results. The left column is the Hough method. The right column is the line segment detector (LSD) method.

We detected line segments in the same image with both the Hough method and the LSD method. The result is shown in Figure 2. It is obvious that many line segments are detected from the tree leaves by the Hough method, which caused many false-negative errors. In contrast, the LSD method detected more accurate and useful line segments, but the Hough method detected shorter and messier line segments than the LSD method. Therefore, the LSD method is better than the Hough method for extracting the line segments needed in order to estimate the location of the road vanishing point.

### 3.2. Removing Invalid Line Segments

After extracting the straight line segments from the road image, some invalid line segments should be removed and do not participate in the voting. These methods are as follows:

1. The line segments of vertical or nearly vertical and horizontal or nearly horizontal orientation (the angle between the line segment and vertical or horizontal orientation is less than or equal to 3°) are removed and are not involved in the voting process. The vertical or nearly vertical line segments are usually the vertical edges of an object (such as a tree trunk, a pole, or a building). The horizontal or nearly horizontal line segments do not point to the vanishing point, so they do not participate in the voting.
2. If the dominant color of the pixel at both ends of a line segment is green, the line segment may be detected from the grass or tree leaves. To determine whether the dominant color of a pixel is green, we define the *Green*(*Z*) function using the color component R, G, B values of the pixel in RGB color space:
  (3) $$Green(Z)=\left\{ \begin{array}{l} 1,\text{ if }(2G_{z}/(R_{z}+B_{z})>T)\text{ and }(G_{z}>R_{z})\text{ and }(G_{z}>B_{z}\text{))}\\ 0\text{ otherwise} \end{array}\right.$$
  where the *R*_z_, *G*_z_, *B*_z_ are the color component *R*, *G*, *B* values of the pixel *Z*(*x, y*). If *Green*(*Z*) = 1, the pixel Z(*x, y*) is green. A lot of tests and experimental results show that this method can better distinguish whether the pixel is green or not when the threshold is *T* = 1.2.
3. Ignoring the line segments on the 1/4 upper region of the image, whose extensional line intersects with the boundary of the image and the two intersection located at the upper part of the 1/3 high of image. These line segments are usually the interfering and invalid lines (such as the line segments detected from the tree leaves and clouds), so they do not participate in the voting.

According to the above methods to remove the invalid line segments, the rests of the line segments are the valid line segments and will participate in the voting for the vanishing point.

### 3.3. Voting Weighting Method

#### 3.3.1. Length Weight

In the line space voting method, the line segments are detected by LSD using the consistent texture orientation. The longer the detected line segment indicates the more pixels in the same direction. Because the long line segments have more of the same directional pixels, they play an important role in the voting process. Therefore, we should give a higher voting weight to the longer line segments to increase their voting score. The voting weight is called the length weight (*W_L_*) which is equal to the length of the line segment divided by the diagonal length of the image. It is defined as

(4) $$W_{L}=L_{line}/L_{D}$$

where, *L_line_* is the length of the original line segment, and *L_D_* is the length of the diagonal length of the image. The longer the line segments the higher the value of *W_L_*.

#### 3.3.2. Orientation Weight

Generally, the directions of the road edge, boundaries, and even ruts and tire tracks left by previous vehicles on the path are often focused on a certain angle range. In the pictures taken by the camera on the vehicle while it is traveling, the direction angles of those lines passing to the vanishing point tend to be near to 45° or 135°, such as the road borders, lane lines, and wheel ruts. These lines are more effective for voting. Therefore, we assign a weight to the lines at different angles, and we call it the orientation weight. In addition, the lines nearly horizontal and vertical are generally invalid lines, which do not pass the vanishing point. As such, they should be removed. Suppose the direction angle of a line is θ(0°~180°), thus the range of absolute value |θ−90°| is 0°~90°. The line whose angle is closer to 45° plays a major role in the voting process. Therefore, the line whose angle is closer to 45° is given the larger orientation weight, and the line whose angle is further away from 45° is assigned the smaller orientation weight. The orientation weight (*W_O_*) is defined as follows:

(5) $$W_{O}=\exp\left(\frac{{\left[abs(\theta -90)-45\right]}^{2}}{-2\times {\left(45\right)}^{2}}\right)$$

**Figure 3:**
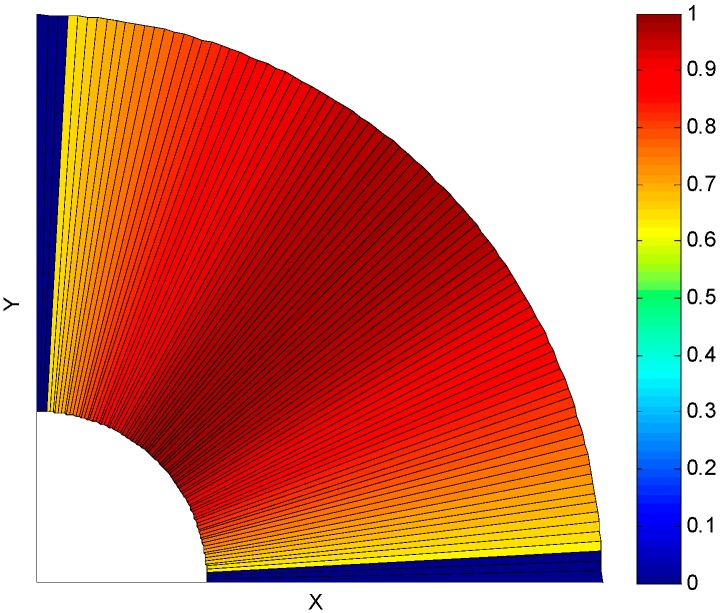
Distribution of the orientation weight in Cartesian coordinates.

The distribution of the orientation weight in Cartesian coordinates is shown as Figure 3. The 0°~90° region is divided into 90 bins. Each bin is a sector with 1°. The line segments with vertical or nearly vertical (0°~3°) and horizontal or nearly horizontal (88°~90°) orientations have been removed in the pre-processing. As such, the orientation weight is 0.

#### 3.3.3. Gaussian Space Weight

**Figure 4:**
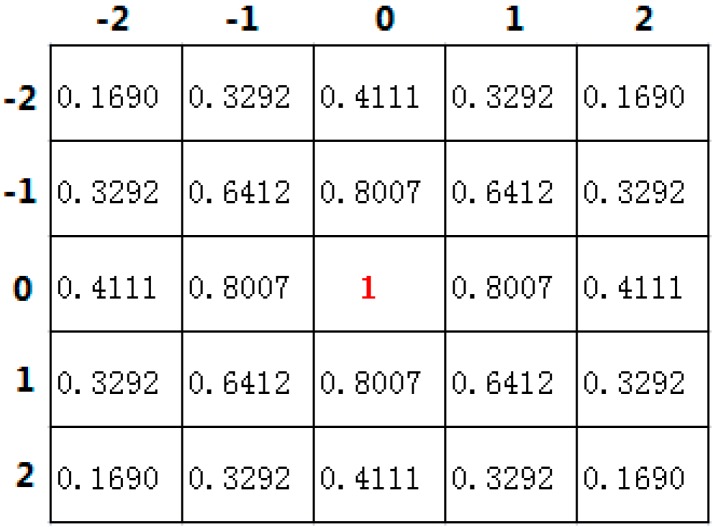
Distribution of Gaussian space weight.

When the points on the line and their neighboring points vote for the vanishing point, we introduce a Gaussian space weight *W_s_* based on the length weight and the orientation weight. In fact, not only the points on the line but also their 5 × 5 neighboring points are involved in the voting process. According to the different positions of the voters, their space weights are assigned different values, which are Gaussian. The aim is to make the lines vote more smoothly, so as to improve the detection precision of the vanishing point. The neighborhood of the points on a line is 5 × 5 matrix space, and the space weight of each point in the neighborhood is calculated by a two-dimensional Gaussian distribution formula as follows:

(6) $$W_{s}(i,j)=\exp\left(\frac{i^{2}+j^{2}}{-2\sigma_{s}^{2}}\right)$$

where the point (*i*, *j*) is in the neighborhood space, $-2\leq i,j\leq 2,\;\sigma_{s}=1.5$, the distribution of the two-dimensional Gaussian space weight $W_{S}(i,j)$ is shown as Figure 4. The Center Point (0, 0) is the point on the line which has the biggest weight. The point which is far away from Center Point has the smaller weight. 

### 3.4. Line Space Voting Scheme

After extracting the line segments from the input image and removing the invalid line segments, a line space voting scheme is used to detect the vanishing point. This voting scheme uses the points on the lines and the points in their 5 × 5 neighborhood space to vote for the VP with the length, orientation, and Gaussian space weight. The method ignores the other pixels, thereby greatly reducing the number of voters, so that it can improve the detection speed of the VP. Moreover, some of these voters have stronger texture orientation pointing to the VP, which are more reliable and effective for voting. The steps of this voting scheme are as follows. First, the remaining line segments are extended to the boundary of the image. Then, we build an accumulator space *M*, which is the same size as the input image and initialized to 0. This accumulator space is used to save the voting score of all the voters. Next, all the voters (i.e., the points on the lines and their neighborhood points) begin to vote for the VP in the corresponding accumulator space. The basic voting score of each voter is set to 1 initially, it is then multiplied by the length, orientation and Gaussian space weight in the voting process. The voting result of a voter is added to the accumulator space of the corresponding position. The voting score of a point (*x*, *y*) on a line and its neighborhood point *M*(*x* + *i*, *y* + *j*) is calculated as follows:

(7) $$M(x+i,y+j)=M(x+i,y+j)+V_{0}(x,y)\times W_{L}\times W_{o}\times W_{s}(i,j)$$

where *V*_0_(*x*, *y*) = 1.0 is the basic voting score, −2≤i,j≤2. The length weight *W_L_*, orientation weight *W_O,_* and Gaussian space weight *W_S_* are calculated by Equations (4)–(6), respectively.

**Figure 5:**
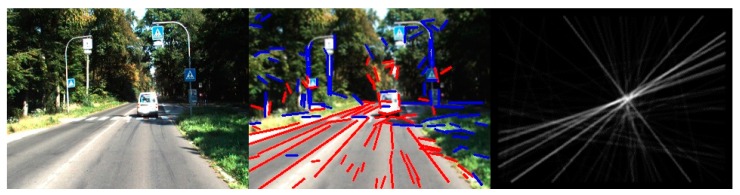
The first column is the input image. The second column is the detected line segments by LSD (the red line segments are valid and the blue line segments are invalid). The third column is the line space voting image.

After all the points on the lines and their neighborhood points finish voting, the accumulator space is smoothed by a Gaussian filter with a 7 × 7 kernel size to get rid of the noise. Finally, the point with the highest score in the accumulator space is considered to be the estimated vanishing point. We normalize the voting score of the accumulator space and obtain the accumulator space map which we call the line space voting image, which is shown in the third column of Figure 5.

### 3.5. Effect of Weights

In Equation (7), we use three weights to vote for the vanishing point. In order to evaluate the effect of these weights on the accuracy of vanishing point detection, we made experiments under different conditions with 30 urban road and highway images with obvious marking lines. The conditions are as follows: (1) *W* = *W_L_* × *W_O_* × *W_S_*, Normal; (2) *W* = *W_O_* × *W_S_*, *W_L_* = 1; (3) *W* = *W_L_* × *W_S_, W_O_* = 1; (4) *W* = *W_L_* × *W_O_*, *W_S_* = 1. We evaluated the position of the vanishing point in these conditions, respectively, and calculated the mean value of the normalized Euclidean distance error (NormDist Error, which is described in Section 4) of the images. The mean value of the NormDist errors and the standard deviation (SD) of the NormDist errors are listed in Table 1, which shows the effect of the weights on the vanishing point detection accuracy. It is obvious that *W_L_* has the largest effect on detection accuracy, and the effect of *W_O_* is minimal.

## 4. Experimental Results and Analysis

In this paper, the proposed algorithm and other relevant algorithms are implemented in Matlab 2014a. All algorithms are evaluated on a laptop computer that has an Intel core i5 2.67GHz processor, 4GB RAM, which is installed with 64bit Win7. In the experiments, we qualitatively and quantitatively evaluate the performance of the proposed method for detecting the vanishing point in unstructured and structured road environments, and we use some state-of-the-art methods to make comparisons in this field. The data set used for the evaluation of the proposed method consists of 600 natural road images. Some are downloaded from the internet by search. Some are from a set of digital photographs taken by DARPA (Defense Advanced Research Projects Agency) Grand Challenge for autonomous vehicles. Lastly, others are from the KITTI Vision Benchmark Suite. In order to ensure the objectivity and impartiality of the evaluation, we select the testing data set which contains 371 unstructured road images in various types of complicated environments (such as desert, mud, snow, shadows) and 229 structured road images (such as urban road, highway). All the images are uniformly resized to the size of 320 × 240 without distortion for fair comparison.

In order to evaluate the accuracy of the proposed algorithm and other algorithms, the vanishing point estimation error was measured by comparing the detected result of all algorithms against the vanishing point ground truth manually determined through human perspective perception. First, we invited seven people in our laboratory to manually mark the vanishing point location in each image from the data set after they are trained to understand the vanishing-point concept. Then, we apply a median filter to the x and y coordinates of these manual estimations, respectively, and the median is used as the initial ground-truth position. After that, the two farthest manually marked locations from the initial ground-truth position are discarded as outliers. Finally, we compute the mean of the other five marked locations, and the result is considered to be the ground-truth vanishing point location. 

To quantitatively measure the vanishing point estimation error, we use the normalized Euclidean distance mentioned in the literature [7]. The normalized Euclidean distance is suitable for different resolution images, which is normalized by the length of the diagonal of the image. The normalized Euclidean distance between the estimated vanishing point and the ground truth is defined as follows:

(8) $$D_{NormDist}=\frac{\left\|P-P_{0}\right\|}{L_{D}}$$

where *P*(*x_p_*, *y*_p_) is the estimated vanishing point, *P*_0_(*x*_0_, *y*_0_) is the ground-truth vanishing point, and *L_D_* is the length of the diagonal of the image. According to this metric, a value closer to 1 indicates that the estimated vanishing point location is further away from the location of the ground truth, and a value closer to 0 means that the estimated vanishing point location is closer to the ground truth. As such, *D_NormDist_* is used to express the error rate of the estimated vanishing point. 

**Figure 6:**
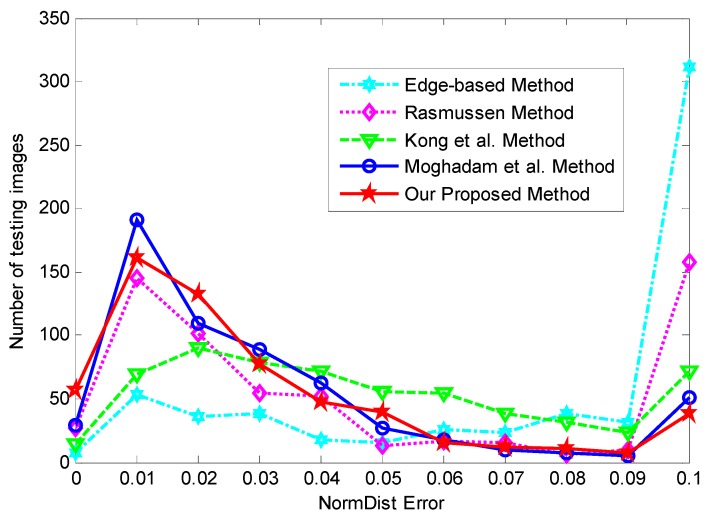
Comparison of vanishing point detection methods in the histogram.

For better qualitative analysis and quantitative comparison, we evaluated the results of the detected vanishing point in off-road and urban environments using the classic edge-based method and three state-of-the-art texture-based methods. We detected the VP from 600 images in the data set using the edge-based method [10], Rasmussen [3] method, Kong et al. [2] method, Moghadam et al. [7] method, and our proposed method, respectively. Then, we evaluated and analyzed the results of these methods in an 11-bin histogram for comparative purposes, as shown in Figure 6. The horizontal axis shows the normalized Euclidean distance (i.e., error rate) between the estimated vanishing point location and the ground truth, and the vertical axis shows the number of testing images in each histogram bin. The distance of 0.01 indicates the error rate is very small, and the distance of 0.1 means the error rate is very large. Therefore, if the error rate is larger than or equal to 0.1, it is placed into the last histogram bin. A larger number of images in the left part of the histogram indicate better results, whereas the larger values in the right part show worse results. It is observed from Figure 6 that the results of the edge-based method [10] are the worst, where about 312 (52%) images have an error rate greater than or equal to 0.1. In the Rasmussen method [3], there are 158 (26.3%) images with error rates greater than or equal to 0.1, and 172 (28.7%) images with an error rate less than or equal to 0.01. The Kong et al. method [2] shows that more than 72 (12%) images have an error rate greater than 0.1 and 84 (14%) images are less than or equal to 0.01. A *D_NormDist_* error that is greater than or equal to 0.1 occurs in only 51 (8.5%) images in the Moghadam et al. method [7], and over 220 (36.7%) images have error rates less than or equal to 0.01. By contrast, our proposed method performs markedly better in that 218 (36.3%) images have error rates less than or equal to 0.01, and only 38(6.3%) images have error rates greater than or equal to 0.1. It is obvious that the performance of our proposed method is better than the edge-based method [10], the Rasmussen method [3], and the Kong et al. method [2], and it is similar to the Moghadam et al. [7] method.

We also evaluated the mean error of these methods in detecting the vanishing point in the testing data set. We categorized all the road images into several categories (desert, mud, snow, shadows, urban road, highway) and we considered the accuracy of every category and the overall accuracy. Table 2 shows the accuracy comparison of the vanishing point detection for every category. The result value of every category is the mean of the normalized Euclidean distance error of each category for each method. The overall mean error is the mean value of the normalized Euclidean distance error of all images for each method. The smaller the mean error, the higher the accuracy of the algorithm.

It is observed from Table 2 that our proposed method is clearly better than other methods in its classifications of the shadows, urban road, and highway categories. However, it is worse than the Moghadam et al. [7] method in its classifications of the desert, mud, and snow categories. Furthermore, the overall performance of our proposed method performs significantly better than the edge-based method [10], the Rasmussen method [3], and the Kong et al. [2] method, and is close to the Moghadam et al. [7] method. The edge-based method has a larger error rate for detecting the vanishing point in the unstructured road environment without obvious road borders or parallel lines, which results in its worse overall performance.

Moreover, the run-time of the proposed method is compared with other methods for evaluating the vanishing points in this paper. The average run-time and the standard deviation (SD) of all compared methods on detecting the vanishing points in the data set are shown in Table 3. A low standard deviation indicates that the run-time tends to be close to the mean of the set, while a high standard deviation indicates that the run-time is spread out over a wider range of values. The mean of the run-time of vanishing point detection for all images is shown in the first row, and the standard deviation of the experimental results is shown in the second row. It is obvious that the run-time of our proposed method is far less than other methods for detecting the vanishing point, which is more suitable for real-time applications. As we know, the frame rate of a camera works well from 20 to 30 frames per second. However, in fact, vanishing point detection does not need to consider all of the frames. In the experiment of vehicle navigation, we let the vehicle run at a speed of 60 km/h with a 30 frame rate camera. It was found that we could get good results when only 8 frames per second were processed for vanishing point detection—that is, the processing time of the vanishing point detection required for vehicle navigation needs 0.125 s. Our proposed method requires 0.14 s to process one frame of image with a Matlab code. After implementing our method with a C++ code, the processing time of the vanishing point detection for one frame was reduced to 0.08 s. As such, our proposed method can meet the real-time requirement for autonomous vehicle navigation.

**Figure 7:**
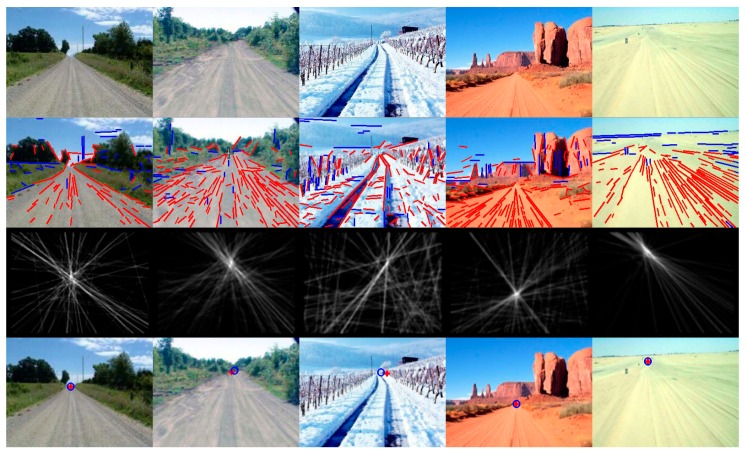
Vanishing point detection and road segmentation examples for unstructured roads using the study’s proposed method. The first row images are the input images. The second row images are the detected line segments. The third row images are the line space voting images. The fourth row images show the estimated VPs and the ground truth (the red cross is the estimated VP location; the blue circle is the ground truth).

**Figure 8:**
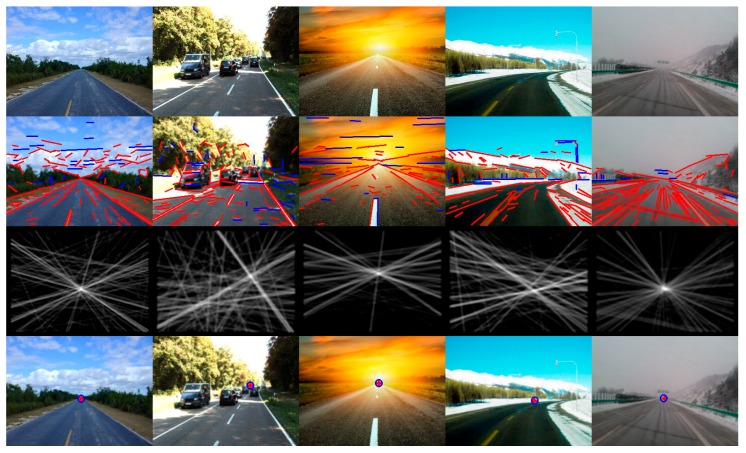
Vanishing point detection and road segmentation examples for structured roads using the study’s proposed method. The first row images are the input images. The second row images are the detected line segments. The third row images are the line space voting images. The fourth row images show the estimated VPs and the ground truth (the red cross is the estimated VP location; the blue circle is the ground truth).

**Figure 9:**
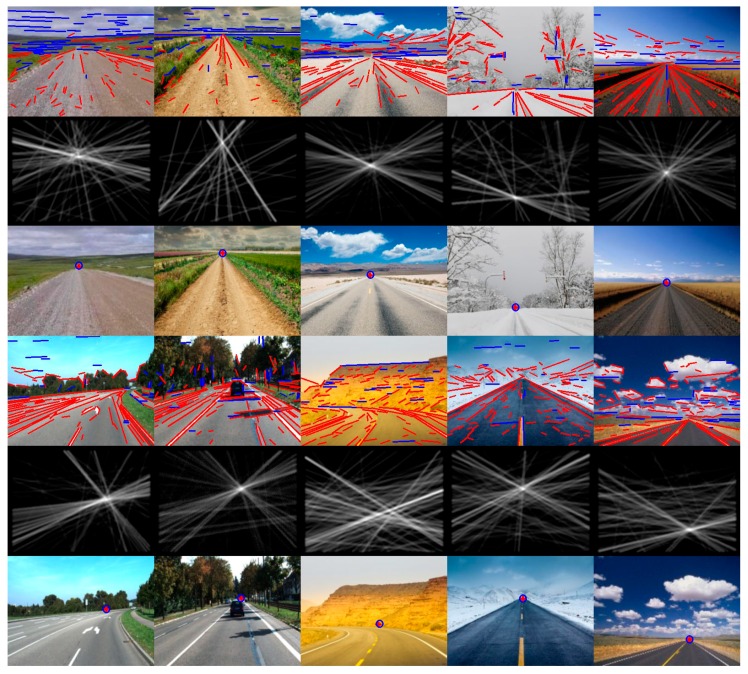
Vanishing point detection and road segmentation examples for different road types using the study’s proposed method. The first and fourth row images are the detected line segments. The second and fifth row images are the line space voting images. The third and sixth row images are the input images covered with the detected VPs.

In order to display the experimental results visually, we list some of the images here. Figure 7 shows some example images for detecting the vanishing point and the road region by using the proposed method, which includes the images in challenging unstructured road environments (such as desert, snow, mud, different illumination conditions). The intersection of the red cross overlaid on the input images is the detected vanishing point location of the road, and the center of the blue circle is the ground-truth vanishing point location. Our proposed method has better adaptability to the structured urban road, because the valid lines can be extracted effectively from the road images, such as those remarkable road edge lines and lane marking lines, which tend to point to the ground-truth vanishing point of the road. Our proposed method is applied to the vanishing point detection and the road region detection in structured road environments, and the experimental results are shown in Figure 8. It is observed from the figure that our proposed method can estimate the vanishing point location of structured roads more accurately. More examples are shown in Figure 9.

## 5. Conclusions

In this paper, we have proposed a novel line space voting scheme for VP detection in general road images. First, the line segments of the road image are detected by the LSD method based on Sobel’s operator which is employed to calculate the gradient and texture orientations. Then, the points of the lines and their neighborhood points vote for the vanishing point in the corresponding accumulator space, and the point that has the highest voting score is considered to be the estimated vanishing point. Compared with other methods, the main contribution of the proposed method is that the algorithm is simple, fast, easy to implement, and has high accuracy. Therefore, the proposed method can meet the real-time requirements of the road recognition for autonomous mobile robots and unmanned vehicles. However, the LSD method may have difficulties detecting the valid line segments in some road images where the road is very smooth or has messy texture direction. This problem will be solved by combining the current proposed method with surface color and texture features in future work.

## Figures and Tables

**Table 1:** The effect of the weights on the vanishing point detection accuracy.

	Normal	*W_L_* = 1	*W_O_* = 1	*W_S_* = 1
NormDist error	0.0181	0.0406	0.0183	0.0218
SD	0.0032	0.0096	0.0058	0.0065

**Table 2:** Accuracy comparison for vanishing point detection.

Road Image Category	Number of Images	Edge-Based Method [10]	Rasmussen [3]	Kong et al. [2]	Moghadam et al. [7]	Our Proposed Method
Desert	104	0.1878	0.0916	0.0532	**0.0365**	0.0403
Mud	90	0.2216	0.1091	0.0863	**0.0654**	0.0913
Shadows	82	0.1128	0.1056	0.0847	0.0722	**0.0685**
Snow	95	0.1062	0.0963	0.0755	**0.0526**	0.0562
Urban road	128	0.0538	0.0515	0.0332	0.0318	**0.0223**
Highway	101	0.0512	0.0502	0.0281	0.0272	**0.0204**
Overall mean error	(600)	0.1181	0.0814	0.0575	**0.0457**	0.0471

**Table 3:** Run-Time Comparison for Vanishing Point Evaluation.

	Edge-Based Method [10]	Rasmussen [3]	Kong et al. [2]	Moghadam et al. [7]	Our Proposed Method
Time(s)	1.58	228.34	86.52	2.36	**0.14**
SD	0.0243	0.0736	0.0415	0.0252	**0.0206**
